# Piloting of WHO Safe Childbirth Checklist using a modified version in Sri Lanka

**DOI:** 10.1186/s13104-018-4009-y

**Published:** 2018-12-14

**Authors:** H. M. Senanayake, M. Patabendige, R. Ramachandran

**Affiliations:** 1 Faculty of Medicine, University of Colombo, Colombo, Sri Lanka; 2 University Obstetrics Unit, Teaching Hospital, Mahamodara, Galle, Sri Lanka; 3 University Obstetrics Unit, De Soysa Hospital for Women, Colombo, Sri Lanka

**Keywords:** WHO, Safe childbirth, Checklist, Implementation

## Abstract

**Objectives:**

Data was gathered to study the impact of a context-specific modified WHO Safe Childbirth Checklist (mSCC) at two tertiary care settings in Sri Lanka, as a part of an implementation program.

**Data description:**

We provide data sets of a prospective observational study which was conducted in the University Obstetrics Unit at De Soysa Hospital for Women (DSHW), Colombo and two Obstetric Units at Teaching Hospital, Mahamodara, Galle (THMG), Sri Lanka. These consist of demographic and checklist implementation details and data on the level of acceptance. The study was conducted over 8 weeks at DSHW and over 4 weeks at THMG. Checklists were kept attached to clinical records at admission and collected on discharge. Level of acceptance was assessed using a self-administered questionnaire. Outcome measures were adoption rate (percentage of deliveries where mSCC was used), adherence to practices (mean percentage of items checked in each checklist), response rate (percentage of staff members who responded to questionnaire) and level of acceptance (percentage of “strongly agree/agree” in Likert scale to five questions regarding acceptance of modified SCC).

## Objective

There are more than one hundred and thirty million births in the world annually. These yield in an estimated 287,000 maternal deaths [1], one million intrapartum stillbirths [2] and three million newborn deaths [3]. Approximately 99% (302,000) of these occur in resource-limited settings and would have been prevented with timely, effective interventions [2, 3]. Substandard care during institutional childbirth in has been recognized as a major contributory factor for childbirth-related harms [4]. Although skilled-attendants may be available in healthcare facilities, they may fail to adhere to accepted protocols due to the failure to remember critical steps and the sequence in which to correctly execute them. A simple checklist that focuses on major causes of maternal mortality and morbidity could overcome these failures [5]. Identifying this need, the World Health Organization (WHO) designed the Safe Childbirth Checklist (SCC) [6, 7]. As recommended by the WHO [8], we included context-specific adaptations in the mSCC in the hope of addressing weaknesses that may have contributed to the low adoption rate in our previous study [9].

This study was conducted to assess if a more context-specific modified SCC (mSCC) would result in an improved adoption rate. The results based on these data has been published in *BMC Pregnancy Childbirth* [10].

## Data description

These data were gathered for a hospital-based, prospective observational study which was carried out in Sri Lanka in the University Obstetrics Unit of De Soysa Hospital for Women (DSHW), Colombo and two Obstetric Units in the Teaching Hospital, Mahamodara, Galle (THMG), two busy tertiary care maternity hospitals in Sri Lanka. Before the introduction of the intervention, the necessary basic education was given to healthcare workers. This consisted of the components of modified mSSC, its relevance to patient safety and quality improvement and how and when to use it. The staff was advised to mark the mSCC items in parallel to the practice of each item, optimizing the value of a checklist in clinical practice. The mSCC was kept attached to clinical notes of every mother from admission to the ward to the point of discharge when they were collected into a separate file. Outcome measures were adoption rate (percentage of deliveries where the mSCC was used during the study period), adherence to practices (mean percentage of each item checked in mSCC out of the total in each setting), response rate and the level of acceptance.

The level of acceptance was assessed using a self-administered, pre-tested anonymous questionnaire at the end of the study period given to all staff involved and a link to a copy of the questionnaire have been provided as in Table 1 [12]. The response rate was the percentage of healthcare providers who responded to this questionnaire. The questionnaire included a five-point Likert scale for five stems focusing on the level of acceptance of SCC use and one open-ended question on the barriers to its use. The answers ‘strongly agree’ and ‘agree’ from the Likert scale were taken as satisfactory levels of acceptance and presented as percentages. Data have been entered in SPSS Spreadsheets and included in Table 1 [11, 12]. Ethical aspects of this study were reviewed and approved by the Ethics Review Committee of the Faculty of Medicine, University of Colombo, Sri Lanka (EC-16-108). Informed written consent was taken from each participant before giving the questionnaire. A copy of the mSCC has been provided as a supplementary file as indicated in Table 1 [13]. It is also available in the study published in *BMC Pregnancy Childbirth* 2018 [10].

**Table 1:** Overview of data files

Label	Name of data file/data set	File types (file extension)	Data repository and identifier (DOI)
Data file 1	Demographic and Checklist data	SPSS file (.sav)	Figshare (10.6084/m9.figshare.7176176.v1)
Data file 2	The level of acceptance	SPSS file (.sav)	Figshare (10.6084/m9.figshare.7176179.v1)
Figure	Questionnaire to assess the level of acceptance	Figure (.PNG)	Figshare (10.6084/m9.figshare.7176179.v1)
Supplementary material	Copy of modified WHO Safe Childbirth Checklist	PDF file (.pdf)	Figshare (10.6084/m9.figshare.7399457)

## Limitations

- This is an observational study without a control group and data was collected from a self-administered questionnaire.
- The data in this study may be more specific to Sri Lanka, where the standard of care is of a better quality compared to most developing countries.
- Looking at checklists that were filled out could overestimate or underestimate its use.
- It is possible that the checklists were simply filled out after delivery or at discharge and not in real time.
- It is also possible that some used the mSCC as a guide, without filling it out.
- Even though authors reinforced their knowledge and attitudes using the Implementation Guide from time to time, this step does not involve a direct unbiased observations.
- When compared to the previous studies from sites in the world which have been conducted with well-planned coaching-based interventions, this study has been conducted with a relatively light-touch intervention.

## Abbreviations

WHO: World Health Organisation
SCC: Safe Childbirth Checklist
DSHW: De Soysa Hospital for Women
THMG: Teaching Hospital Mahamodara, Galle
mSCC: Modified Safe Childbirth Checklist
